# The Treatment of Insanity: Existing Defects and their Remedy

**Published:** 1907-05-04

**Authors:** 


### THE TREATMENT OF INSANITY.

### Existing Defects and their Remedy.

### IV. WHERE ALIENISM IS LAMENTABLY WANTING.

No physician would dispute the statement that our knowledge of the pathology of insanity, and our methods of treatment of insanity, have not kept pace with the advance of knowledge and treatment in other departments of medicine. The treatment of the insane from a humanitarian point of view has, indeed, advanced much more than the treatment of other diseases, more even than the treatment of criminals; but then it had more leeway to make up. We do not now chain our lunatics to the floors, the walls, the tables and the bedposts; we do not keep them naked on straw, wallowing in their own filth; we do not flog them periodically as a curative measure; any more than we bleed or purge our patients with small-pox, or than we hang our criminals by the score for petty theft. We have nothing now to reproach ourselves with on the score of want of kindness and consideration for these unfortunates, but if we ask what measures are taken to cure them of the grevious malady with which they are afflicted, the account that must be given is less satisfactory.

Complaint is made not merely that the treatment of insanity is not successful, but that the methods and appliances that are brought to bear upon the investigation of this disease are not such as have proved so successful in other departments of medicine, and are not in accordance with modern discoveries and modern requirements; that the spirit and the methods of modern scientific pathology have never been applied to research in insanity; that all the efforts of those who have care of the insane are concentrated upon making them comfortable, while the curative treatment is neglected. This is the charge that we propose to examine.

Now, it must be declared at once that the failure of alienists to discover a cure for insanity is no reproach to them whatever. It is vain to point to the achievements of other men in other departments of medicine, and to draw a contrast to the disadvantage and discredit of the alienist. It is quite true that there has been, in the treatment of insanity, no such astonishing leap forward as there has been in the treatment of wounds, in the treatment of hydrophobia, of diphtheria, of rheumatism, of tetanus, of ague, of plague, and of many other maladies; but, on the other hand, it is to be remembered that with all our improvements of method, with all the refinements of instruments, and with all our diligence of research, we have not advanced a single step in the treatment of cancer, and scarcely a step in our knowledge of its nature. The problem of insanity may be as insoluble as the problem of cancer; and it is no reproach, no discredit to the alienist, that he has not solved a problem that may, for aught we know, turn out to be insoluble. But the alienist is not entitled to a verdict on this ground unless and until he can show that he has exhausted all the resources of science in his efforts to find a remedy. He must be judged not by his results, but by the efforts that he has made to arrive at a result; and in this it is that alienism is so lamentably wanting.

In every other department of medicine investigation is continually proceeding; in a score of directions efforts are made to penetrate into the recondite processes of the living body, and to discover and measure and correct their aberrations. How much of all this is done in lunatic asylums? It is true that there is a pathological laboratory at Claybury which works for the whole of the London county asylums; and there is another such laboratory maintained by a number of Scottish asylums; and here and there a small amount of work is done in a sporadic manner in a few of the many asylums that are scattered over the country. In one place clinical investigation is carried on with zeal for a time by one worker; and in the laboratories that have been mentioned, pathological research has been carried on by other workers; but there is not, and has never been, that co-ordination of clinical observation with research work in the clinical and pathological laboratories which obtains in every large general hospital, and to which the splendid advances are due that have been recorded in other departments of medicine.

The complaint is an old one, and various efforts have been made to meet it. Recently built asylums—and of these there are many—have been in many cases furnished with a "hospital annexe" for the reception and treatment of recent and acute cases; and this addition to the buildings of the asylums has been hailed with extreme satisfaction, and regarded as a sufficient answer to those who complained that the scientific investigation of insanity is neglected in lunatic asylums. It is "hospital treatment" that is required, and therefore, so it seems to have been argued, if we build an annexe and call it a hospital, and house our patients therein, the demands of the grumblers will be met, and all that is required of us will be done. Alas! If insanity could be cured by expenditure on bricks and mortar, or by calling old things by new names, it would be the very foremost, instead of the most backward, branch of medicine. The "hospital annexe" has been no more productive of discoveries in pathology and treatment than the old "refractory wards." The "hospital annexe" contains much the same mixture of all sorts of cases as the rest of the building, and these cases are treated by the same medical officers in the same way and with the same results. Nothing is changed, except that more money has been spent and more buildings erected. But where a thoroughly equipped laboratory has been instituted, with a thoroughly able man at the head of it, and a sufficient staff, have the results been such as to warrant or encourage an extension of the system? Unquestionably they have. Very excellent work has been done both at Claybury and at Edinburgh, and striking and important results obtained.

